# Benign Osteonecrosis of the External Auditory Canal: Diagnostic Challenges and Outcomes in a Case Series

**DOI:** 10.7759/cureus.109737

**Published:** 2026-05-27

**Authors:** Milad Golsharifi, Nita R Rajan, Solomon Pudi

**Affiliations:** 1 Otolaryngology, Tameside General Hospital, Manchester, GBR

**Keywords:** aural debridement, benign osteonecrosis, bisphosphonate, denosumab, external auditory canal, medication-related osteonecrosis, necrotising otitis externa, retrospective study, tympanic plate

## Abstract

Introduction

Benign osteonecrosis (BON) of the external auditory canal (EAC) is a rare and under recognised otological condition characterised by localised bony necrosis of the tympanic plate. It represents a diagnostic challenge due to its clinical overlap with necrotising otitis externa (NOE), EAC malignancy, keratosis obturans, and other external ear pathologies. Risk factors include antiresorptive pharmacotherapy, particularly bisphosphonates and denosumab, hearing aid use, local mechanical trauma, haemodialysis, and smoking. This retrospective case series aims to characterise the clinical presentation, investigative findings, management strategies, and outcomes of patients diagnosed with BON at a single UK ENT centre.

Methods

A retrospective review was conducted of seven patients diagnosed with BON of the EAC at the ENT Outpatient Clinics, Tameside General Hospital, between January 2023 and December 2025. Inclusion criteria required a confirmed diagnosis based on clinical examination, histopathological biopsy, microbiological culture, and high-resolution computed tomography (HRCT) of the temporal bones. Data on patient demographics, presenting symptoms, risk factors, investigations, management, and follow-up outcomes were collected and analysed.

Results

Seven patients (four male, three female; median age 67 years, range 55-80) were included. None had diabetes mellitus. Three patients (43%) had received bisphosphonate therapy for three to five years. Three patients used hearing aids. One patient had chronic kidney disease requiring haemodialysis, and one presented with bilateral disease. All patients underwent HRCT temporal bone imaging and histopathological biopsy. Biopsy confirmed chronic inflammation and bony sequestrum without malignancy or features of NOE in all cases. All seven patients were managed conservatively with serial aural debridement and topical antimicrobial therapy. Progressive epithelialisation was achieved in all patients over a follow-up period of three to eight months. No patient required surgical intervention or hyperbaric oxygen therapy.

Conclusions

BON of the EAC is an underreported condition that can be successfully managed conservatively in the majority of cases. Clinician awareness is essential, particularly in patients receiving antiresorptive agents or using hearing aids. Exclusion of malignancy and NOE through biopsy and HRCT imaging is mandatory. Further prospective research is required to establish standardised diagnostic criteria and management guidelines.

## Introduction

Benign osteonecrosis (BON) of the external auditory canal (EAC) is a rare otological condition characterised by localised avascular necrosis of the tympanic plate, in the absence of malignancy or primary infective aetiology. The condition was first described by Wolf et al. in 1997, who attributed it to idiopathic vascular insufficiency combined with local trauma [1]. Since then, a growing number of case reports and small series have expanded understanding of its pathogenesis, risk factors, and clinical spectrum [2-5].

The pathophysiology of BON is believed to centre on disruption of the periosteal blood supply to the tympanic plate, resulting in avascular necrosis [1,2]. Predisposing factors include systemic conditions that impair microvascular perfusion, such as diabetes mellitus, chronic kidney disease (CKD), and smoking, as well as local mechanical trauma from hearing aid use or cotton-tipped applicator insertion [2,3]. Antiresorptive pharmacotherapy, particularly bisphosphonates and the receptor activator of nuclear factor kappa-B (RANK) ligand inhibitor denosumab, has emerged as a significant aetiological factor. Bisphosphonates inhibit osteoclast-mediated bone resorption, impair vascular endothelial growth factor (VEGF) signalling, and suppress bone remodelling, creating conditions analogous to medication-related osteonecrosis of the jaw (MRONJ) [4-7]. More recently, denosumab has been independently implicated in medication-related osteonecrosis of the EAC (MROEAC), even without prior bisphosphonate exposure [8,9].

BON presents a substantial diagnostic challenge. Its clinical appearance, with exposed necrotic bone, otorrhoea, and bony canal defects, overlaps significantly with necrotising otitis externa (NOE), EAC squamous cell carcinoma (SCC), keratosis obturans, and EAC cholesteatoma [2,10]. Unlike NOE, which typically causes severe pain and carries a risk of intracranial spread, BON is often minimally symptomatic relative to the degree of tissue involvement [3,11]. No universally accepted diagnostic criteria or validated staging system currently exists for BON.

Despite increasing recognition of BON in the literature, published series remain limited, and the condition is likely underdiagnosed. Khan's 2024 rapid review highlighted a significant lack of standardised management pathways and called for greater awareness among ENT surgeons and prescribers of antiresorptive agents [6]. This retrospective case series contributes to the existing evidence base by presenting the clinical profiles, investigative findings, management strategies, and outcomes of seven patients diagnosed with BON at a single UK ENT centre.

## Materials and methods

Study design and setting

This was a retrospective observational case series conducted at the ENT Outpatient Clinics, Tameside General Hospital, Ashton-under-Lyne, United Kingdom. The study period spanned January 2023 to December 2025.

Inclusion and exclusion criteria

Patients were included in the study if they met all of the following criteria: Presentation to the ENT Outpatient Clinic with symptoms referable to the EAC, including otorrhoea, otalgia, aural fullness, or reduced hearing; Clinical and microscopic findings demonstrating a bony EAC defect with exposed or necrotic bone; Histopathological biopsy of the canal defect confirming chronic inflammation and bony sequestrum, without evidence of cellular dysplasia, malignancy, or granulomatous features of NOE; high-resolution computed tomography (HRCT) temporal bone imaging demonstrating localised bony erosion of the EAC wall without intracranial extension or discrete soft-tissue mass; Microbiological culture results available to exclude primary infective aetiology.

Patients were excluded if any of the following applied: Histopathological evidence of malignancy or squamous cell carcinoma of the EAC; Radiological or clinical features consistent with NOE, including cranial nerve palsy, skull base erosion, or intracranial extension on imaging; Incomplete clinical documentation or failure to attend follow-up appointments.

Data collection

Patient records were retrospectively reviewed. Data extracted included: age, sex, presenting symptoms, duration of symptoms, otoscopic and microscopic examination findings, relevant past medical history, current medications (with specific attention to antiresorptive agents and hearing aid use), laterality of disease, microbiological culture results, histopathological findings, HRCT temporal bone findings, management undertaken, and clinical outcomes at final follow-up.

Investigations

All patients underwent the following standardised investigations: otoscopic and microscopic examination of the EAC; punch biopsy of the canal defect for histopathological analysis and microbiological culture; HRCT of the temporal bones with thin-slice axial and coronal reconstructions; serum inflammatory markers (C-reactive protein, erythrocyte sedimentation rate); and fasting blood glucose and HbA1c to exclude diabetes mellitus. Full blood count, renal function, and liver function tests were performed in all patients. Immunological screening was undertaken where clinically indicated.

Primary and secondary outcomes

The primary outcome of this study was the rate of epithelialisation and healing of the EAC bony defect, as assessed by clinical and microscopic examination at follow-up appointments, in patients managed conservatively with serial aural debridement and topical antimicrobial therapy.

Secondary outcomes included: (i) the proportion of patients requiring escalation of treatment beyond conservative management (i.e., surgical intervention or hyperbaric oxygen therapy); (ii) the duration of follow-up required to achieve clinical resolution; (iii) identification of risk factors associated with BON in this cohort; and (iv) the microbiological and histopathological profile of the disease in this patient population.

Management protocol

All patients were managed initially with conservative measures. Serial micro-suctioning and aural debridement of necrotic bony debris were performed at interval outpatient appointments. Topical antibiotic-steroid preparations, including ciprofloxacin-hydrocortisone drops and Terra-Cortril ointment, were prescribed. Patients with secondary infection were treated with culture-directed topical and, where appropriate, oral antimicrobial therapy. Patients using hearing aids were counselled to switch to softer ear moulds. Those receiving bisphosphonate therapy were referred for specialist physician review regarding the risk-benefit balance of continuing antiresorptive treatment.

## Results

Patient demographics

Seven patients were included in the study (four male, three female). The median age was 67 years (range 55-80 years). All patients were of White British ethnicity. No patient had a confirmed diagnosis of diabetes mellitus. A summary of patient demographics, clinical findings, risk factors, and outcomes is provided in Table 1. Patients are labelled A through G.

**Table 1:** Summary of patient demographics, clinical findings, risk factors, and management outcomes. BP: bisphosphonate; BCC: basal cell carcinoma; CKD: chronic kidney disease; COPD: chronic obstructive pulmonary disease; DLP: dyslipidaemia; DM: diabetes mellitus; EAC: external auditory canal; F/U: follow-up; HA: hearing aid; HTN: hypertension; IHD: ischaemic heart disease; LVH: left ventricular hypertrophy; PMH: past medical history; TM: tympanic membrane.

Patient	Age	Sex	Symptoms	EAC findings	DM	HA	BP use	Duration of BP use	PMH	Side	F/U (mo)	Management
A	80	M	Ear discharge, no pain	Floor of canal defect with necrotic bony debris extending to TM	No	Yes	No	N/A	COPD, HTN, IHD, BCC pinna	Left	5	Conservative: serial micro-suctioning
B	78	F	Ear discharge, reduced hearing	Anteroinferior defect with skin break and necrotic debris	No	Yes	Yes	Three years	HTN, COPD, DLP, Smoking	Right	8	Conservative: serial micro-suctioning
C	63	M	Earache, discharge, reduced hearing	Posteroinferior canal defect with skin loss and necrotic bone	No	No	No	N/A	None	Left	3	Conservative: serial micro-suctioning
D	64	M	Earache, reduced hearing	EAC cholesteatoma with anteroinferior canal defect and necrotic bone	No	No	No	N/A	CKD (haemodialysis)	Left	3	Conservative: serial micro-suctioning
E	77	F	Pressure sensation, ear discharge	Floor of canal defect with necrotic bone and skin loss	No	Yes	Yes	Four years	Osteoporosis, HTN, LVH	Right	3	Conservative: serial micro-suctioning
F	62	F	Reduced hearing	Anteroinferior canal defect with necrotic bone and skin loss	No	No	Yes	Five years	Osteoporosis	Bilateral	6	Conservative: serial micro-suctioning
G	55	M	Ear discharge, earache	Floor of canal defect with necrotic bone near TM	No	No	No	N/A	None	Left	6	Conservative: serial micro-suctioning

Clinical presentation

The most common presenting symptoms were ear discharge (otorrhoea; five patients), otalgia (three patients), and reduced hearing (three patients). One patient (patient E) presented primarily with aural pressure sensation. Symptom duration prior to presentation ranged from four weeks to six months. All patients had visible bony EAC defects on otoscopic and microscopic examination, with exposed or necrotic bone at the floor or anteroinferior wall of the canal. One patient (patient D) had concurrent EAC cholesteatoma.

Risk factors

Three of seven patients (43%; patients B, E, and F) had received bisphosphonate therapy for a duration of three to five years prior to presentation. Hearing aid use was documented in three patients (patients A, B, and E). One patient (patient D) had chronic kidney disease requiring haemodialysis. One patient (patient B) had a documented smoking history. No patient was receiving denosumab at the time of diagnosis. Four patients had no exposure to antiresorptive agents, suggesting that BON can arise in the context of non-pharmacological risk factors alone.

Investigations

**Figure 1:**
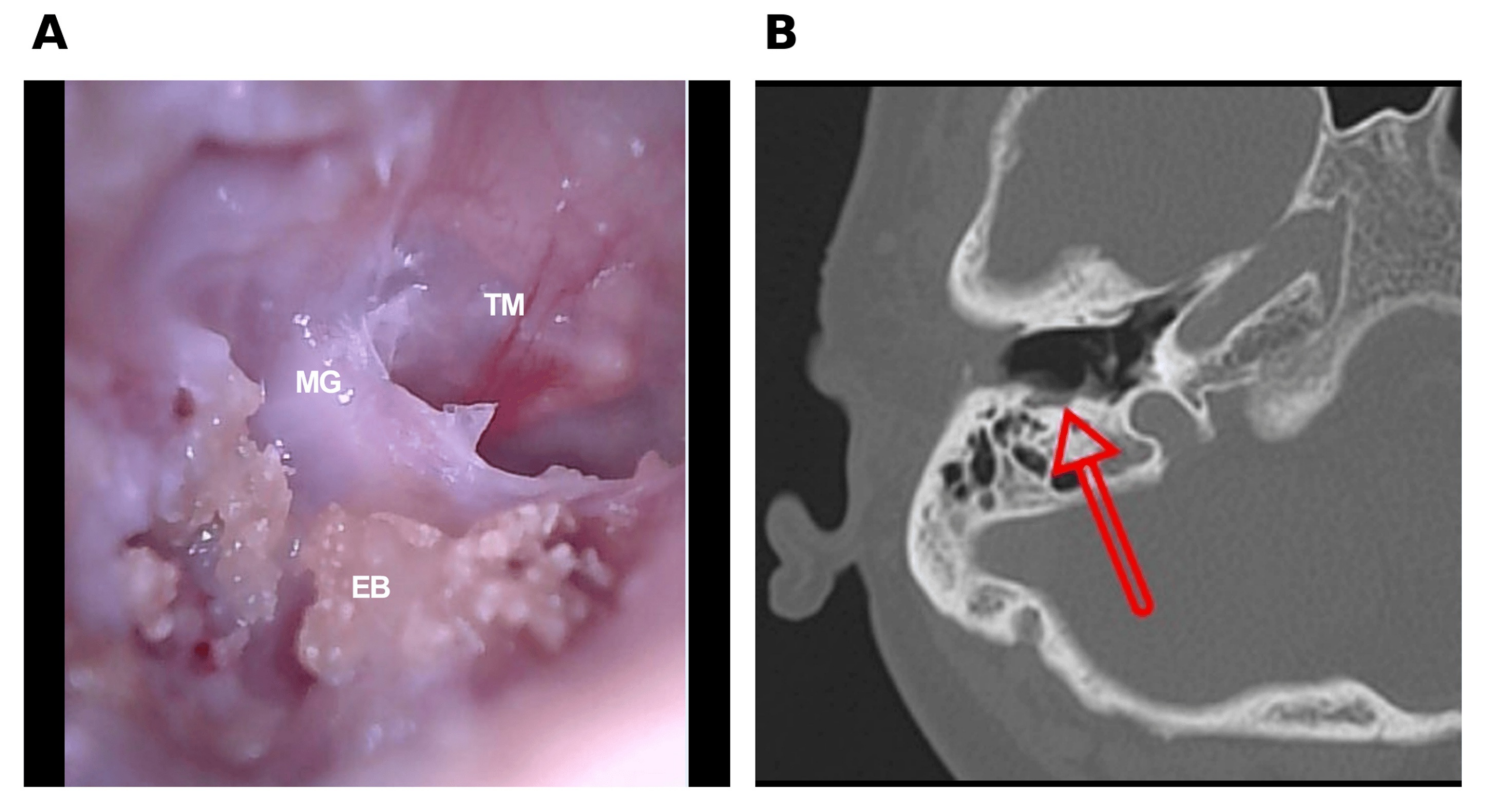
Radiological and endoscopic findings in patient E with benign osteonecrosis of the external auditory canal. (A) Endoscopic clinical image of the EAC showing partial mucosalisation of the inferior indentation and bone exposure. (B) Axial CT of the temporal bone at the level of the inferior EAC, demonstrating indentation of the inferior EAC wall (arrow). TM: tympanic membrane; MG: membrane growth; EB: exposed bone; EAC: external auditory canal.

Histopathological biopsy confirmed chronic inflammation and bony sequestrum in all seven patients, with no evidence of cellular dysplasia, malignancy, or NOE in any case. Microbiological cultures were positive in four patients, most commonly yielding *Staphylococcus aureus *and *Pseudomonas aeruginosa*, consistent with secondary colonisation rather than primary infection. HRCT temporal bone imaging in all patients demonstrated localised bony erosion of the inferior or anteroinferior EAC wall without evidence of a discrete soft-tissue mass, middle ear or mastoid involvement, intracranial extension, or cranial nerve involvement. Representative imaging and endoscopic findings for patient E are illustrated in Figure 1. Inflammatory markers were mildly elevated in two patients. No patient had serological evidence of immunocompromise, and none met biochemical criteria for diabetes mellitus.

Treatment and outcomes

All seven patients were managed conservatively. Progressive epithelialisation of the bony EAC defect was achieved in all patients, representing a primary outcome success rate of 100%. The duration of follow-up required to achieve clinical resolution ranged from three to eight months (median five months). No patient required surgical intervention or hyperbaric oxygen therapy. Patients A, B, and E were advised to adopt softer hearing aid ear moulds. Bisphosphonate therapy was reviewed by the prescribing physician in patients B, E, and F; continuation was determined on an individualised risk-benefit basis in each case. No patient experienced disease recurrence within the study follow-up period.

## Discussion

This retrospective case series adds to a growing body of literature documenting BON of the EAC as a clinically distinct and under recognised entity. Our cohort of seven patients from a single UK ENT centre published to date demonstrates that conservative management is effective in achieving full epithelialisation of bony defects across a heterogeneous group of patients with diverse risk factor profiles. The primary outcome, clinical resolution with conservative therapy, was achieved in all seven cases.

The pathogenesis of BON centres on microvascular compromise to the periosteum of the tympanic plate. In patients receiving bisphosphonates, suppression of osteoclast-mediated bone turnover and impairment of VEGF-dependent angiogenesis create a milieu analogous to MRONJ [4-6,12]. Three of our patients (43%) had received bisphosphonates for three to five years, consistent with published series demonstrating a dose-duration relationship [5,6]. McCadden et al. reported six cases of bisphosphonate-induced EAC osteonecrosis with similar conservative outcomes [5], Salzman et al. described the histopathological and radiological features in bisphosphonate-associated cases [4], and Wickham et al. reported osteonecrosis of the EAC associated with bisphosphonate use, presenting two cases with differing antiresorptive exposures [13]. Polizzotto et al. further documented bisphosphonate-associated osteonecrosis of the auditory canal, corroborating the causal relationship between antiresorptive therapy and bony necrosis [7]. Whilst none of our patients received denosumab, Kumar et al. and True et al. have each documented MROEAC attributable to denosumab, including bilateral cases [8,9], highlighting that the spectrum of culpable antiresorptive agents extends beyond bisphosphonates.

The complete absence of diabetes mellitus in our cohort is a notable finding. Diabetes has historically been regarded as a key risk factor for EAC osteonecrosis, drawing a parallel with the pathophysiology of NOE [11,14]. Our data, consistent with those of Whittaker and McKinnon [2] and Chandler et al. [3], suggest that BON frequently occurs in non-diabetic patients, with antiresorptive pharmacotherapy and local mechanical trauma serving as equally or more significant aetiological drivers. This distinction has important clinical implications: BON should not be discounted in patients without diabetes, and a thorough medication history is essential.

Four patients in our cohort had no exposure to antiresorptive agents, reinforcing that BON can arise from non-pharmacological mechanisms alone. This is consistent with the original description by Wolf et al. [1], suggesting that localised vascular insufficiency and mechanical microtrauma may be sufficient to precipitate disease in susceptible individuals.

The diagnostic workup in this series followed a systematic protocol aligned with published guidance [2,3,15]. Histopathological biopsy was central to excluding EAC malignancy, which may present with an indistinguishable clinical appearance. HRCT temporal bone imaging was essential for characterising the extent of bony erosion, excluding middle ear or mastoid involvement, and differentiating BON from NOE. The absence of a discrete soft-tissue mass, intracranial extension, or cranial nerve involvement on imaging in all seven patients was consistent with the benign, localised nature of BON. The mild elevation of inflammatory markers in two patients likely reflected secondary bacterial colonisation rather than primary infective aetiology, as supported by microbiological culture results.

Conservative management with serial micro-suctioning, aural debridement, and topical antimicrobial-steroid therapy was effective in all seven patients. This is consistent with the predominant experience reported in the literature [2,3,5,16]. Surgical management, including sequestrectomy, canal plasty, and fascial flap reconstruction, as described by Saxby and Linder [16], remains reserved for refractory or advanced cases. Hyperbaric oxygen therapy has been proposed as an adjunct to promote revascularisation in resistant disease [17], though this was not required in any of our patients. The counselling of hearing aid users to adopt softer ear moulds represents a simple but clinically important measure to reduce ongoing mechanical trauma to the EAC epithelium.

Limitations

This study has several limitations that should be acknowledged. First, the retrospective, single-centre design limits the generalisability of the findings. Selection bias may have influenced the cohort, as only patients presenting to a specialist ENT clinic were included, potentially excluding those with milder disease managed in primary care. Second, the sample size of seven patients, whilst representing a meaningful contribution to the limited published literature, is insufficient to draw statistically robust conclusions regarding risk factor associations or the superiority of any specific management strategy. Third, the follow-up duration of three to eight months, whilst sufficient to demonstrate clinical resolution in all patients, may not be adequate to capture late recurrence or disease progression. Fourth, the study lacks a control group, precluding formal comparative analysis. Fifth, microbiological culture results and antibiotic sensitivities were not consistently documented across all patients, limiting the characterisation of the infective microenvironment. Finally, the absence of validated patient-reported outcome measures (PROMs) is a limitation; future studies should incorporate standardised instruments to capture the impact of BON on quality of life. Despite these limitations, this series contributes meaningful clinical data to an underrepresented area of otological literature and provides a framework for future prospective multicentre research.

## Conclusions

Benign osteonecrosis of the EAC is an underreported and diagnostically challenging condition that can successfully mimic both benign and malignant external ear pathologies. A systematic diagnostic approach incorporating otoscopic examination, histopathological biopsy, microbiological culture, and HRCT temporal bone imaging is essential to exclude malignancy and NOE and to confirm the diagnosis. This retrospective series of seven patients demonstrates that conservative management, comprising serial aural debridement and topical antimicrobial therapy, achieves complete epithelialisation in all cases, with no requirement for surgical intervention.

Clinicians should maintain a high index of suspicion for BON in patients presenting with chronic otorrhoea and focal bony EAC exposure, particularly those receiving antiresorptive pharmacotherapy or using hearing aids. Patients on long-term bisphosphonate or denosumab therapy who have additional risk factors for EAC trauma should receive targeted aural hygiene education and be referred promptly for ENT review if new otological symptoms develop. Prescribers of antiresorptive agents should be aware of MROEAC as a potential complication, analogous to the established risk of MRONJ. Further prospective, multicentre research is needed to establish standardised diagnostic criteria, a validated staging system, and evidence-based management guidelines for BON of the EAC. The growing body of literature, of which this series forms a part, supports the recognition of BON as a distinct clinicopathological entity warranting dedicated clinical and research attention.
